# Host personality and seasonal parasitism risk do not account for egg rejection behavior in the azure-winged magpie

**DOI:** 10.1016/j.ijppaw.2025.101056

**Published:** 2025-03-19

**Authors:** Xingyi Jiang, Wei Liang, Yanyun Zhang

**Affiliations:** a Ministry of Education Key Laboratory for Biodiversity and Ecological Engineering, College of Life Sciences, Beijing Normal University, Beijing, 100875, China; b Ministry of Education Key Laboratory for Ecology of Tropical Islands, Key Laboratory of Tropical Animal and Plant Ecology of Hainan Province, College of Life Sciences, Hainan Normal University, Haikou, 571158, China

**Keywords:** Brood parasitism, Cuckoo parasitism, Egg rejection, Host personality, Nest defense

## Abstract

Brood parasitism reduces the reproductive success of hosts and many host birds have evolved a range of anti-parasitism strategies, including egg recognition and egg rejection. Recent studies have shown that host egg rejection behavior can vary according to personality traits and parasitism risk. However, these relationships have not been clearly determined. The aim of this study was to further investigate the influence of seasonal parasitism pressure and host personality traits on egg rejection behavior in the azure-winged magpie (*Cyanopica cyanus*). Our results showed no significant difference in the proportion of egg rejection between hosts with low (before the arrival of cuckoos) and high (after the arrival of cuckoos) parasitism pressure. In addition, no significant difference was detected in the proportion of egg rejection between bold individuals (shorter flight initiation distance, FID) and shy individuals (longer FID). We hypothesized that the relatively weak effect of the presence or absence of cuckoos on this species could be attributed to their inherently strong egg recognition abilities. Moreover, the quantification of host behavior along a single personality axis (boldness-shyness) may be insufficient to capture behavioral differences that arise from the combined effects of various personality traits. Our study provides novel insights into the influence of seasonal parasitism risk and personality traits on host egg rejection behavior.

## Highlights

• In the magpie-cuckoo parasitic system, the high egg rejection by the azure-winged magpies (*Cyanopica cyanus*) indicates a coevolutionary history with parasitic cuckoos.
• Neither the presence of cuckoos (i.e., the risk of cuckoo parasitism) nor the boldness-shyness personality axis of the magpies was correlated with egg rejection behavior.
• Our study provides new evidence regarding the influence of seasonal parasitism risk and personality traits on host egg recognition and rejection behavior.

## Graphical abstract

There was no significant difference in egg rejection rate between Azure-winged Magpies (*Cyanopica cynaus*) experimentally parasitized before the arrival of cuckoos and those experimentally parasitized after the arrival of cuckoos.In addition, no significant difference was detected in the proportion of egg rejection between bold magpie individuals (shorter flight initiation distance, FID) and shy individuals (longer FID).The quantification of host behavior along a single personality axis (boldness-shyness) may be insufficient to capture behavioral differences that arise from the combined effects of various personality traits.

**Figure:**
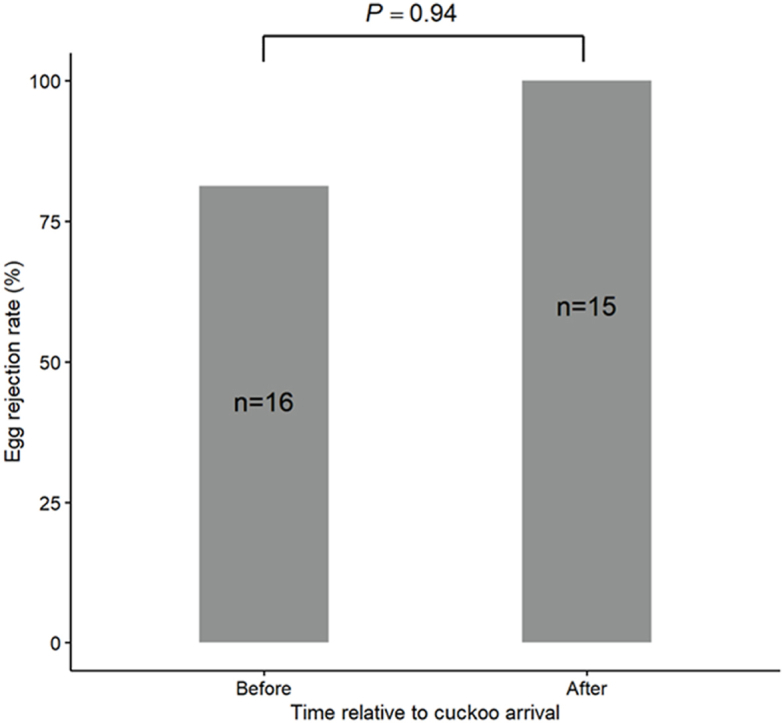

## 1 Introduction

Brood parasitism serves as a model system for studying the evolution of species interactions (Feeney et al., 2012). Parasitic cuckoos (*Cuculus* spp.) impose the cost of nurturing offspring on hosts by laying their eggs in the nests of other species, which reduces the reproductive success of the host (Davies, 2000). The costs incurred by the host and the benefits gained by the cuckoo contribute to a coevolutionary arms race of deception and recognition across different reproductive stages (Kilner and Langmore, 2011). For example, some host species can recognize adult cuckoos and respond by mobbing or attacking (Welbergen and Davies, 2009; Abernathy and Liang, 2020). Conversely, cuckoos mimic the appearance (Thorogood and Davies, 2012) and calls (York and Davies, 2017; Zhang et al., 2021) of sparrowhawks (*Accipiter* spp.) to avoid attack and increase their chances of successful parasitism. In many cases, research has focused on the egg and nestling stages of this evolutionary arms race (Feeney et al., 2012). While host birds often lack the ability to recognize chicks (e.g., Grim, 2006; Soler, 2009; Yi et al., 2020; but see Yang et al., 2015), many hosts have evolved the ability to detect, recognize, and reject foreign eggs (Davies, 2000; Soler, 2017). Host defense at the egg stage may mitigate some reproductive losses to a certain extent, however, several cuckoo species will remove at least one host egg during laying (Davies, 2000) and some damage eggs directly by pecking at them (Astié and Reboreda, 2006; Spottiswoode and Colebrook-Robjent, 2007).

Hosts can rely on a variety of cues to distinguish and reject foreign eggs, such as visual features, including egg color and spotting (e.g. Feeney et al., 2014; Hanley et al., 2017; Wang et al., 2020). In contrast, in breeding environments with inadequate lighting, such as closed nests, hosts may be more likely to rely on tactile features (Mason and Rothstein, 1986; Tosi-Germán et al., 2020; Ye et al., 2023). Additionally, cues such as olfactory signals (Soler et al., 2014) and memory of the spatial arrangement of eggs (Polačiková et al., 2013) have also proven to be effective. Accordingly, cuckoos can mimic host egg characteristics to hinder host detection (Davies, 2011; Stoddard and Stevens, 2010). When dealing with hosts that exhibit egg color polymorphism, cuckoo females may selectively parasitize based on eggshell appearance, a strategy known as the “egg matching” hypothesis (Davies, 2000). For example, Daurian redstarts (*Phoenicurus auroreus*) lay blue or pink eggs, and common cuckoos (*Cuculus canorus*) have a tendency to lay their eggs in nests with similar egg color (Zhang et al., 2023; but see Yang et al., 2016a, Yang et al., 2016b, 2017).

Parasitism risks and selection pressures differ across environments and time scales, and this variation may drive differences in the ability to recognize eggs among various host populations (Thorogood and Davies, 2013; Liang et al., 2016). For example, in a population of Daurian redstarts in northern China, the proportion of individuals rejecting eggs was significantly higher during the second breeding peak of the season than during the first peak. This increase can be attributed to the arrival of common cuckoos in the breeding area and the onset of parasitism only during the second breeding peak of the year (Zhang et al., 2021a). However, in a similar seasonal migratory system involving the Isabelline shrike (*Lanius isabellinus*) and common cuckoo, no significant difference in the rejection rates of foreign eggs was observed despite seasonal changes in parasitism risk (Zhou and Liang, 2024). In addition, the host personality hypothesis suggests that host personality traits influence defense against parasitism (Avilés and Parejo, 2011). However, host personality traits remain a prominent gap in avian brood parasitism research (Yang, 2021), with only one study clearly showing that personality traits can predict host egg rejection behavior (Zhang et al., 2021b; but see Shen et al., 2023).

In this study, the azure-winged magpie *(Cyanopica cyanus*) was used to test whether individuals with different personalities exhibit different anti-parasitism behaviors under various parasitism pressures. Specifically, we first tested the egg rejection in azure-winged magpies with different personalities (bold and shy) by focusing on the boldness–shyness axis (reflecting an individual's response to risk). We then compared differences in egg recognition and egg rejection of the azure-winged magpie before and after the arrival of cuckoos (representing weaker and stronger seasonal parasitism risks, respectively). Based on the host personality hypothesis, we predicted that bold azure-winged magpies would be more likely to reject foreign eggs. Additionally, due to the increasing parasitism risk, we expected the egg rejection behavior of the azure-winged magpies to increase following the arrival of migratory breeding cuckoos at the study site.

## 2 Materials and methods

### 2.1 Study area and study species

The study was conducted in April–June 2024. The study site was located in Fusong County, Baishan City, Jilin Province, northeastern China (42°42ʹ N, 127°01′ E). The region is located in the temperate zone with an average elevation of 481 m, a continental monsoon climate with cold and snowy winters, and an average annual temperature of 4 °C (Yang et al., 2019).

**Fig. 1:**
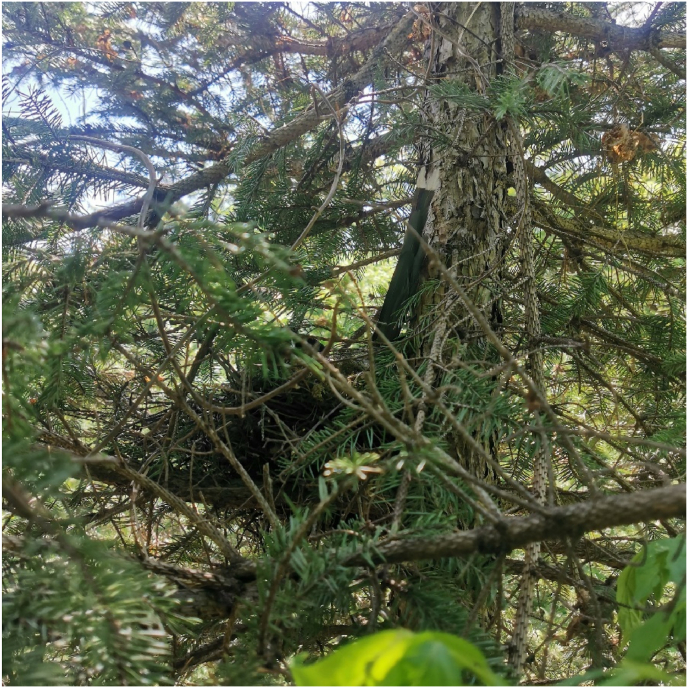
Image of the nest and an azure-winged magpie incubating eggs.

Azure-winged magpies belong to the order Passeriformes and family Corvidae. They are widely distributed in the eastern Palearctic region and exhibit cooperative breeding behavior. The helpers assist with courtship feeding, provisioning the nestlings, and defending the nest (Ren et al., 2016). Azure-winged magpies serve as an important host for the common cuckoo in Japan (Nakamura, 1990), while in China, they are parasitized by common cuckoos, Indian cuckoos (*Cuculus micropterus*) (Nakamura et al., 1998), and Asian koels (*Eudynamys scolopaceus*) (Lin et al., 2024). They are typical resident species in most parts of China (Zheng, 2023). However, at our study site, they appeared in surrounding coniferous forests near populated areas only around April, and primarily used sporadically distributed, artificially planted spruce (*Picea asperata*) and red pine (*Pinus koraiensis*) woods for nesting (Liu et al., 2023). Nests were usually located on branches of the main trunk of coniferous trees, where dense sharp needles form natural protection (Fig. 1; Video S1).

### 2.2 Data collection and field experiments

During the breeding season, we searched for natural nests of the azure-winged magpie daily and checked nests every two days to determine and record the breeding status. When egg laying was complete, we initiated the escape behavior test and a foreign egg recognition experiment (on the first day of incubation).

We quantified the personality traits using flight initiation distance (FID), which is defined as the distance between an individual and a predator when escape behavior is initiated (Blumstein, 2003). FID can be used to represent the level of boldness of an individual (e.g., Ducatez et al., 2017; Himes and La Valle, 2023; Shen et al., 2023). Individuals with a longer FID were identified as relatively shy, whereas those with shorter FID were regarded as bolder. Incubation is carried out by females, so the personalities of males were not considered in our study.

The researcher first observed the incubating female through a telescope from a distance of 5 m from the nest tree, determining the natural head orientation of the incubating female. This direction was used to define the front of the nest. Due to the shelter provided by the dense needles, the female would not be disturbed in this distance. The researcher then approached the nest tree from either side (perpendicular to the female's head orientation) to stand at the area directly below the side of the nest. In all cases, the incubating female remained in the nest and did not fly away but do show some vigilance like looking around during this procedure. Then, the researcher used a retractable carbon fiber pole (0.8–3 m in length) with a marker tied to the front end to simulate a predator. The pole was moved vertically upward from below at a constant speed until the incubating individual flew away. When the incubating bird (e.g., the female, Video S1) took flight, we marked the tip of the pole on the nearest branch and measured the distance from this point to the nearest outer edge of the nest using a measuring tape. As all incubating females left the nest and flew out of our sight, we were able to confirm that they did not witness the egg experiment.

**Fig. 2:**
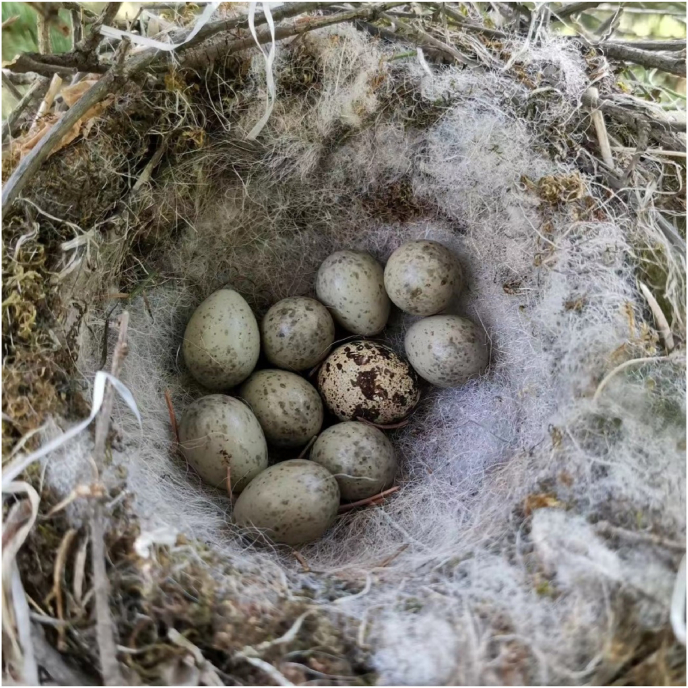
A clutch of nine azure-winged magpie eggs and one experimental Japanese quail egg.

After completing the measurements, we placed a commercially available unfertilized Japanese quail (*Coturnix japonica*) egg in the nest before the incubating female returned, while avoiding the effect of the bird witnessing the egg experiments (Hanley et al., 2015). Japanese quail eggs are non-mimetic eggs, slightly larger than those of the azure-winged magpie, with spots distributed on the surface (Fig. 2). Previous studies have evaluated egg recognition and egg rejection in azure-winged magpies using quail eggs (Liu et al., 2023). We set the egg rejection experiment period to 6 days (counting the day the egg was introduced as day 1). If the introduced egg remained in the experimental nest and no nest abandonment occurred within the 6-day period, the female in the experimental nest was considered to not reject foreign eggs (Liu et al., 2023). Clutch size and nest height (the distance from the outer bottom of the nest to the ground) were also recorded. Since a full breeding cycle takes approximately 2 months, including one month of post-fledging care (Gao et al., 2018), and no new active nests were recorded after June, we assume that no pair bred more than once during the experimental period from April to July. In other words, all pairs were tested during their first breeding attempt of the year.

Cuckoos distributed in the region consisted primarily of common cuckoos and Indian cuckoos, which typically arrive at breeding sites in mid-May (Zhang et al., 2021a). We first recorded the presence of the common cuckoo at the study site on May 7, along with its calls on the same day. Based on this, we categorized the nests into groups depending on whether the egg experiments concluded before or after May 7, 2024 to investigate whether seasonal changes in egg rejection rates corresponded with the seasonal variation in cuckoo parasitism risk.

We did not include a control treatment to separate the potential effects of the FID experiment on host decisions. Although predation risk may influence the egg ejection rate (Roncalli et al., 2019), we applied equal artificial predation pressure to each nest, as all experiments were conducted by XJ in consistent clothing. Therefore, the impact of the FID experiment on egg recognition (if it exists) should be consistent across all nests. Blinded methods were not used. It was not possible to record data blind because our study involved focal animals in the field, the experimenter had to know how to measure FID of the focus nest and which nests to experimentally parasitize.

### 2.3 Data analysis

We converted the observed FID into categorical variables to reduce the variability of this measure. We used k-means clustering to identify natural groupings of the actual distance. Silhouette widths were calculated for each clustering solution (K = 2 to K = 5), and the clustering solution with the highest silhouette coefficient (K = 3, Silhouette Coefficient = 0.64) was selected. Based on clustering and FID values, the personality categories were labeled as Shy, Intermediate, and Bold. These personality labels were then used in the subsequent analyses.

We first used a Generalized Linear Mixed Model (GLMM) to analyze whether the personality of female azure-winged magpies and the presence of cuckoos influence their egg rejection behavior. The rejection behavior (rejection = 1, acceptance = 0) was the response variable, and the fixed effects included personality categories (Shy, Intermediate, Bold), the interaction with cuckoo presence (i.e., whether the experiment was conducted before or after the cuckoo arrived), and clutch size, with nest height as a random effect. Since the interaction effect was not significant (estimate = −0.214, SE = 9629, z = 0.000, *P* = 1.000), and the contribution of nest height as a random effect was negligible (variance = 0.0004), we simplified the model to avoid overfitting, given the small sample size. The final model used a Generalized Linear Model (GLM) with a binomial distribution to analyze factors influencing egg rejection, retaining only the main effects of FID, cuckoo presence, and clutch size. Statistical analyses were performed using R version 4.3.1 (R Core Team, 2023).

## 3 Results

We found 48 natural nests of azure-winged magpies, of which only 32 nests were subjected to egg experiments due to predation of the parent birds or encounters with nest predation before or during egg laying. One nest experienced predation after the introduction of a foreign egg, making it impossible to determine whether the host rejected the egg. Consequently, this nest was excluded from further analyses. No natural parasitism was observed in the nests we found.

**Fig. 3:**
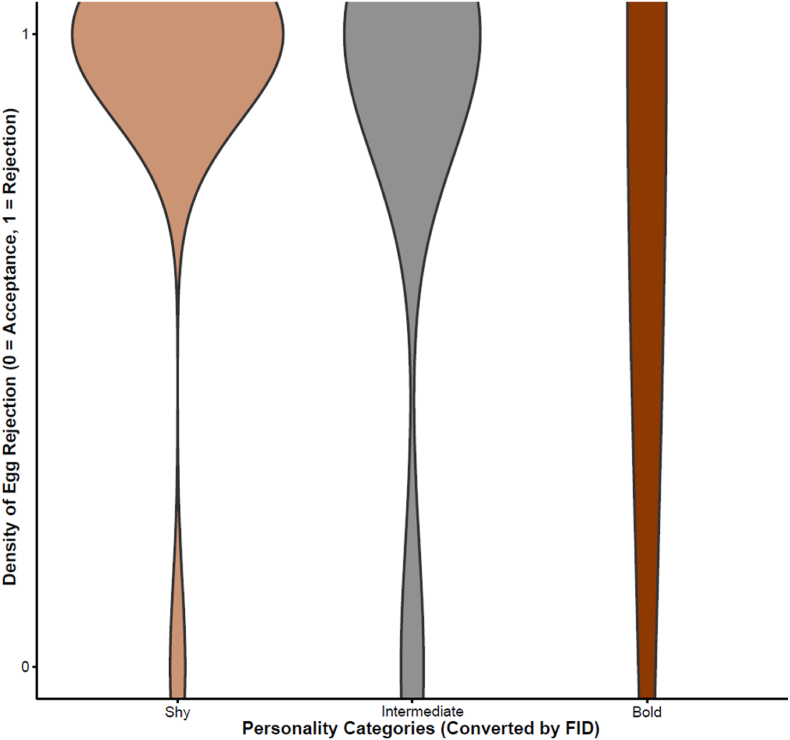
Density of quail egg rejection and personality category (converted by FID) of azure-winged magpie.

**Fig. 4:**
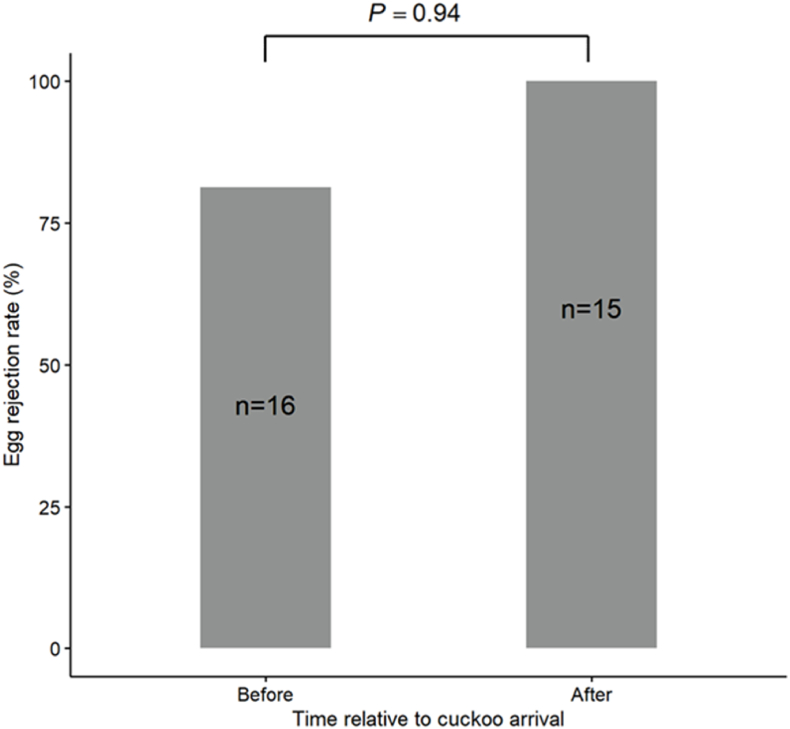
Comparison of quail egg rejection rates by azure-winged magpies before and after the arrival of cuckoos.

In the 31 nests where the egg rejection experiments were conducted, 90.32 % of the individuals rejected the foreign egg. GLM results indicated that clutch size had no significant effect on the egg rejection rate (estimate = 0.39, SE = 0.68, z = 0.58, *P* = 0.57). Before the arrival of the cuckoos, the host egg rejection rate was 81.2 % (13 out of 16 nests). After the arrival of the cuckoos, all tested individuals successfully rejected the foreign eggs (15 out of 15 nests). However, the GLM results showed that the presence of the cuckoo did not significantly affect the egg rejection rate (estimate = −19.67, SE = 4775.3, z = −0.004, *P* = 0.997; Fig. 3). The personality of hosts did not affect their egg rejection rate (Personality-Intermediate: estimate = −0.780, SE = 1.361, z = −0.573, *P* = 0.567; Personality-Bold: estimate = −0.066, SE = 13390, z = 0.000, *P* = 1.000; Fig. 4).

## 4 Discussion

Our results show that azure-winged magpies effectively recognize and reject foreign eggs, regardless of cuckoo presence at the breeding site, personality traits of the host and clutch size. Previous studies have shown that azure-winged magpies effectively reject foreign eggs by recognizing their own (Liu et al., 2023, 2024). Although no cases of azure-winged magpies parasitized by cuckoos were found in our study, we observed a high rate of egg rejection (90.3 %). Similarly, the Iberian magpie, a species closely related to the azure-winged magpie, demonstrates high efficiency in recognizing the eggs of its primary parasite, the great spotted cuckoo (*Clamator glandarius*; with an egg rejection rate of 73.7 %) (Avilés, 2004).

The history of parasitism in azure-winged magpies is relatively short. In Japan, parasitism by common cuckoos began about 40 years ago and defensive behaviors against cuckoos were initially rare (Nakamura, 1990). However, as the rate of cuckoo parasitism increased rapidly, the frequency of magpies mobbing cuckoos and rejecting eggs also rose sharply over a few decades (Nakamura et al., 1998). It has been proposed that the rejecting behavior increased too rapidly to be a result of a genetic change (Davies and Welbergen, 2009). Instead, researchers suggest that the hosts learned to respond to cuckoos and exhibit a pre-existing, but phenotypically flexible, egg rejection behavior (Nakamura et al., 1998). In other words, the ability to recognize and eject eggs may have been part of this population's genetic makeup, evolving long ago and being retained due to its low cost (Avilés, 2004). The flexibility of egg rejection behavior across populations or timelines could be influenced by social learning and communication system (Soler, 2011). However, the social transmission mechanism may not serve as the main explanation in this study, since it is unlikely that the azure-winged magpies in Fusong population have experienced cuckoo parasitism, as no natural parasitism was observed over at least three years. Although social learning may not directly enhance egg recognition abilities in this population, it could interact with the evolved anti-parasitism mechanisms, contributing to the high egg rejection rates observed in this population. For example, hosts may become aware of cuckoo presence from social information, which could favor their defenses (Thorogood and Davies, 2016). Cooperative behavior may also play a role in nest defense (Wang et al., 2023). As a colonial cooperative breeder, azure-winged magpies built open, bowl-shaped nests, with the shortest distance between adjacent nests being 2.3 m in our population. Therefore, it is possible that helpers, neighbors, and other young roamers contribute to nest defense by monitoring active nests (Soler, 2011). In terms of communication systems, although these birds can provide specific information about different predation events (Jiang and Zhang, 2024), there is no empirical evidence that they associate specific vocalizations with brood parasitism. This remains a challenging question that may warrant further investigation.

In contrast to results of the Daurian redstart (Zhang et al., 2021a), we did not observe a significant difference in the ability of azure-winged magpies to recognize and reject foreign eggs before and after the arrival of cuckoos. Similar results have been reported in Isabelline shrikes. Although the shrikes displayed increased aggression as the season progressed, their egg rejection rate did not change (Zhou and Liang, 2024). Unlike Daurian redstarts, which lay at least two clutches per year (Zhang et al., 2021b), we did not observe a second breeding attempt by the azure-winged magpie, and no new nests were found after June, indicating relatively few breeding opportunities. The parental re-nesting potential declines rapidly during the breeding season (Barash, 1975), which may increase the relative benefits of egg rejection behavior when reproductive opportunities are limited. Therefore, consistently high egg rejection rates will help maximize the return on breeding investment and ensure breeding success. The relative intensity and seasonal fluctuations in parasitism risk as well as changes in re-nesting potential may result in egg rejection behavior in populations with no apparent overall seasonal variation.

In addition, we did not find a significant effect of the boldness–shyness personality axis on magpie egg rejection behavior. Studies of the role of the boldness–shyness personality axis in various species have yielded inconsistent results (e.g., Trnka and Grim, 2014; Zhang et al., 2021b; Shen et al., 2023). These inconsistencies may suggest that the effects of personality on egg rejection behavior are species-specific and shaped by multiple factors. For instance, unlike the monomorphic eggs of the azure-winged magpie, the population of Daurian redstarts has both pink and blue colored eggs, and the egg rejection ability differs with respect to color type (Yang et al., 2016a, Yang et al., 2016b; Zhang et al., 2023). Thus, it is also possible that the seasonal variation in egg rejection rates of Daurian redstarts (Zhang et al., 2021a) and the high rate of egg rejection in bold individuals (Zhang et al., 2021b) could be the result of different proportions of individuals with different egg recognition abilities in the population. Unlike the personality studies mentioned above, we did not conduct repeated personality measurements for each individual. This decision was partly made to avoid the risk of causing reproductive failure in cold weather. On the other hand, numerous studies have shown that FID is a relatively stable and repeatable personality trait (e.g., Møller, 2014; Hammer et al., 2022), especially when there are no significant changes in the surrounding environment (Cabrera et al., 2017). Nonetheless, there is no published study has directly tested the repeatability of FID in our population, which is a limitation of the present study. Despite the non-significance of our findings, it does not imply that personality plays no role in the anti-parasitic defenses of the azure-winged magpie. Animals have diverse personality traits (Réale et al., 2007); accordingly, testing a single personality axis may not be sufficient to reflect behavioral differences. For example, for social birds, the sociability of the host individual, another personality trait, may be more likely to influence the risk of parasitism and thus lead to changes in egg rejection rates (Yang and Feeney, 2022). In some cases, socialized individuals may show greater susceptibility to brood parasitism than solitary breeding individuals because the additional activity of conspecifics in the vicinity of the nest may attract the attention of the parasite (Poiani and Elgar, 1994; Nahid et al., 2024). Nevertheless, the presence of conspecifics could also enhance defenses in socially breeding individuals, helping to prevent brood parasitism (Poiani and Elgar, 1994; Feeney et al., 2013).

In the azure-winged magpie-cuckoo parasitic system, the high egg rejection by magpies indicates a coevolutionary history with parasitic cuckoos. However, neither the presence of cuckoos (i.e., the risk of cuckoo parasitism) nor the boldness-shyness personality axis of the magpies was correlated with egg rejection behavior. While studies have shown that host age and experience can influence egg recognition, especially in long-lived species (Lotem et al., 1992; Martínez et al., 2020), it is challenging to accurately age adult azure-winged magpies based on plumage or morphometrics. As a result, we were unable to incorporate host age into this study. Future studies should explore the role of host age, the presence and number of helpers on host egg rejection behavior and its defense mechanisms to further improve our understanding of the relationship between host sociality and anti-parasitism adaptations towards cuckoos.

## CRediT authorship contribution statement

**Xingyi Jiang:** Writing – original draft, Validation, Methodology, Investigation, Formal analysis. **Wei Liang:** Writing – review & editing, Supervision, Resources, Conceptualization. **Yanyun Zhang:** Writing – review & editing, Supervision, Funding acquisition, Conceptualization.

## Data accessibility

The datasets used for this study (Data Table S1) and supplementary material (Video S1) are available at https://figshare.com/s/e732c910b37ece2fd291 (https://doi.org/10.6084/m9.figshare.27019630).

## Ethical approval

This study was approved by the Ethic and Animal Welfare Committee, College of Life Sciences, Beijing Normal University on animal ethics (approval number: CLS-EAW-2021–020).

## Funding

This work was supported by the 10.13039/501100001809National Natural Science Foundation of China (grant nos. 32170516 to YZ and 32270526 to WL).

## Declaration of interest

The authors declare that they have no known competing financial interests or personal relationships that could have appeared to influence the work reported in this paper.
